# Medical News

**Published:** 1853-02

**Authors:** 


### MEDICAL NEWS.

Dr. J. B. Biddle has been appointed lecturer on Materia Medica and Therapeutics in the *Philadelphia Medical Institute.*

From the well established reputation and success of Dr. Biddle as a teacher, we predict increased popularity to the school with which he is connected.

Dr. Bernard Henry has been appointed to the chair of Physiology in the same school. S.

Singular Investigations in a Trial for Murder.—The following curious case has excited great attention in the law courts of Berlin:

Sept. 10, 1849, some peasants found on the bank of a rivulet, which flowed into the Spree, the body of a man, whose head had been detached by an incision carried between the first and second dorsal vertebræ. The head had been so much disfigured by the assassins that recognition was impossible. Near the body was a small cane, a hat, and a box of allumettes; some of the clothes were remaining on the trunk.

The day following two physicians drew up a report, which was unsatisfactory and imperfect. Some time afterwards they both in court declared, together with the magistrates present at the examination, in answer to some questions, that there were no cicatrices from scarifications, nor marks of tattooage upon the body.

A girl came forward, and stated, that from the accounts published in the Berlin journals, she felt sure the deceased was her husband. The body was disinterred, and she recognized it by the external organs of generation, as well as by the clothes. Yet the witness was found to be a prostitute, who never had been married in her life. Other researches led to the supposition, that the assassin of the unknown individual was a cattle merchant, named Gottlieb Ebermann. These suspicions, however, did not last long, for there came reason to believe, that Ebermann was the man murdered. It was said of him, that he might be recognized by traces of the cupping scarificator on the wrists, and tattoo marks of a heart and of the initials "G. E" on the left arm, both of which points of identification were asserted by the very surgeons who had bled him. But Ebermann's sisters and wife stated, that they knew nothing of such marks, consequently there was a second exhumation, five months after the death, but no traces were found on the body. The wife's evidence was not considered valuable, as she had been only recently married and much separated from her husband. A person then came forward, and declared that he had seen and spoken to Ebermann within four-and-twenty hours; he, however, was proved a madman. Lastly, a mistress of Ebermann stated positively that the little cane found near the body belonged to a man of small stature, once a postilion, now a brigand, named Schall, at whose lodgings Ebermann's own cane had been seized. The girl recognized the dress, and particularly the braces, which she had herself worked. There was a third exhumation, December 11, 1851, twenty-six months after the death, when the girl recognized the body by something peculiar in the teeth and the beard. On August 11, 1851, this same girl had been nearly assassinated; doubtless by the accomplices of Schall, then in prison. The question as to whether the scarifications and the tattoo marks, seen upon Ebermann's body by competent witnesses, could by possibility become effaced by time, was referred to M. Caspar of Berlin. In his report, taken from the observations made in a large asylum for aged and invalid soldiers, a class upon whom tattoo marks are common, he states that out of 36 examples, in 3 the tattooage had become faint with time; in 2, the marks were partially effaced; in 4, they were completely obliterated: consequently, says M. Caspar, the marks of tattooage can disappear. A witness came forword, and declared, during the investigation, that at 15 he had tattooed himself on the arm with cinnabar, and that the marks had become entirely effaced. The conclusion of the trial was, that Schall was condemned to death.

In *L'Union Médicale*, Nov. 16, 1852, Dr. Chereau justly observes, respecting Caspar's report, that it is not one which should influence a judicial decision, for it is not stated at what age, with what substance, and in what manner, the marks were produced in the four instances where there was complete obliteration. Are the men to be trusted? How many years elapsed before the marks became effaced? The question cannot be considered in any way satisfactorily settled as it now stands; indeed, Caspar's assertions tend to raise doubts, which heretofore did not exist, upon a point which might be most important in a prisoner's favor, viz., the persistence of these stains. There is evidence that the absorbent glands in the neighborhood of a tattoo mark become filled with pigment. At the time of writing this report, there is in the dissecting-rooms attached to St. Bartholomew's Hospital, the body of a native of one of the islands of the Eastern Archipelago, whose skin has been ornamented to an extent but rarely seen. The whole back, from the sacrum to the shoulder, is covered with circles, radiating stars, and feathers; the arms and the thighs are both marked, but the front of the body is comparatively clear. The absorbent glands in the groin and about the axilla were of deep black hue; those in the neck of the ordinary white color. Mr. Coote, the demonstrator of anatomy, succeeded in dissecting out some absorbent vessels leading to the glands in the thigh, filled with black pigment in long streaks. These indications of the action of the absorbents were however few, and the tattoo marks existed everywhere with as much clearness apparently as at the time when they were first made.

A similar remaık may be offered respecting the possibility of the disappearance of cicatrices. If there have been a complete loss or division of integument in its whole thickness, the mark remains obvious till decomposition after death destroys the tissues. If the skin be only partially destroyed, there ensues a cicatrix of a different kind; one much more resembling the natural structure of the skin, unattended with contractions, and capable of becoming very faint, and liable to be overlooked, except upon close examination. We have no hesitation of expressing our opinion that Caspar's report does not tend in any way to invalidate the statement which has heretofore been received in courts of law—namely, that tattoo marks and cicatrices are indelible.—*London Med. Times and Gaz.*

[The following Communication was accidentally omitted in the Original Department. It is therefore introdueed in this place.—Eds.]

The Modus Operandi of Acids in Scurvy. By Wm. H. White, M. D.—In presenting the following views to the notice of the medical public, it is not so much the object of the writer to enter into detail as to the symptoms and appearance of the disease termed "Scorbutus," as it is to advance a few ideas relative to what he believes to be the state of the blood and the modus operandi of acids and vegetables containing acids, and to shew forth the reason why, if possible, it is, that these articles should, and do operate beneficially in the cure of this disease.

The disease *itself* has been treated of by nearly all writers on medicine since about the middle of the sixteenth century. And all have advanced, in their different essays, the same idea relative to the state of the circulating fluid. They speak of it as if there were a deficiency of some of its constituent parts, which is supplied to the blood by the acid administered for the cure of the disease, and the deficiency being removed, the patient is restored to health.

That the seat of the disease rests entirely in the blood, is admitted on all sides; and that medicines prove of little benefit unless at the same time acids or fresh vegetables be administered, is as unanimously admitted.

But the ground upon which we wish to differ in opinion from those invariably expressed by different writers is, that there is actually *no* deficiency in the blood, but on the other hand an excess, and that excess an alkaline principle which successfully opposes the taking place of that chemical change which the blood should undergo in order to be of a proper chemical constitution. This alkaline principle (soda or potash) exists in the blood in excess, owing to the patient having been deprived of the food from which the system might obtain acid in order that it be neutralized, and the salts of the blood be formed.

If we examine the blood taken from the arm of a scorbutic patient, it will be seen to have undergone considerable chemical change, which is manifested by its very dark appearance, and its being in a completely dissolved state,—not coagulating unless there has been inflammation present.

Now, should we take such blood as soon as drawn from the arm, and add to it an acid, it will assume its natural appearance and color, and will coagulate the same as that taken from the arm of a healthy individual. In like manner will the acid operate upon the blood of a scorbutic patient when given him, by its being taken into the circulation and restoring the *blood* to health—not by the blood taking and assimilating a portion of the acid to itself merely,—but by its producing a chemical change in the blood: The acid, in its round with the circulation after it has been absorbed, comes in contact with the alkaline principle (soda or potash) which is in excess, and neutralizes it and forms the salts of the blood. The system having been previously deprived of the material from which it might obtain the acid to produce this change, the alkalies have been allowed to increase to such an excess as to produce the disease. And the very moment the patient has administered to him the acid or the food containing it, he commences rapidly to advance towards the stage of perfect recovery.

If we were to credit one class of writers we would be led to believe that the disease consisted in an excess of "fibrine," and on the other hand, to credit a second class, that it consisted in a *deficiency* of fibrine and an excess of red corpuscles. But we believe the most rational conclusion is, that the blood possesses alkaline principles in excess, and the acid, by neutralizing them, forms the salts to which the blood is legitimately entitled.

*Philadelphia, Jan.* 1853.

